# Drones as a tool to study and monitor endangered Grey Crowned Cranes (*Balearica regulorum*): Behavioural responses and recommended guidelines

**DOI:** 10.1002/ece3.10990

**Published:** 2024-02-13

**Authors:** Carmen R. Demmer, Stuart Demmer, Trevor McIntyre

**Affiliations:** ^1^ Department of Life and Consumer Sciences University of South Africa Johannesburg South Africa; ^2^ KwaZulu‐Natal South Africa

**Keywords:** breeding, disturbance effects, on‐foot monitoring, protocol, unmanned aerial systems

## Abstract

Crane populations are declining worldwide, with anthropogenically exacerbated habitat loss emerging as the primary causal threat. The endangered Grey Crowned Crane (*Balearica regulorum*) is the least studied of the three crane species that reside in southern Africa. This data paucity hinders essential conservation planning and is primarily due to ineffective monitoring methods and this species' use of inaccessible habitats. In this study, we compared the behavioural responses of different Grey Crowned Crane social groupings to traditional on‐foot monitoring methods and the pioneering use of drones. Grey Crowned Cranes demonstrated a lower tolerance for on‐foot monitoring approaches, allowing closer monitoring proximity with drones (22.72 (95% confidence intervals ‐ 13.75, 37.52) m) than on‐foot methods (97.59 (86.13, 110.59) m) before displaying evasive behaviours. The behavioural response of flocks was minimal at flight heights above 50 m, whilst larger flocks were more likely to display evasive behaviours in response to monitoring by either method. Families displayed the least evasive behaviours to lower flights, whereas nesting birds were sensitive to the angles of drone approaches. Altogether, our findings confirm the usefulness of drones for monitoring wetland‐nesting species and provide valuable species‐specific guidelines for monitoring Grey Crowned Cranes. However, we caution future studies on wetland breeding birds to develop species‐specific protocols before implementing drone methodologies.

## Graphical abstract

Grey Crowned Crane social groupings showed greater tolerance to drone monitoring, displaying evasive behaviours at 23 m compared with 98 m when monitored on foot. The behavioural response of flocks was minimal at flight heights above 50 m, whilst larger flocks were more likely to display evasive behaviours in response to monitoring by either method. Families displayed the least evasive behaviours to lower flights but we caution that nesting birds are sensitive to drone approach angle.

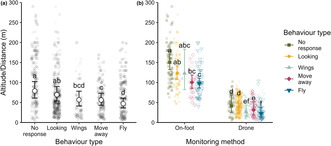

## 1 INTRODUCTION

Wetlands are among the most efficient and diverse ecosystems globally (Balwan & Kour, 2021). They play a crucial role in controlling climate change, sustaining the global hydrological cycle, conserving biodiversity and improving human well‐being (Kingsford et al., 2016; Mitsch et al., 2015; Nováková & Robovský, 2021; Xu et al., 2019). Despite their importance, almost 50% of wetlands have been lost globally, thereby negatively affecting biodiversity and ecosystem functioning (Xu et al., 2019). Effectively implementing monitoring programs is essential for safeguarding the remaining wetland ecosystems and their distinct contributions (Bal et al., 2018; Lindenmayer & Likens, 2011; Malhi et al., 2020; Williams et al., 2021). Birds often play a key role in these efforts, serving as reliable ecological indicators due to their well‐established research history, widespread distribution across various habitats, and predictable responses to environmental changes (Fraixedas et al., 2020).

Cranes belong to the *Gruidae* bird family and commonly act as ambassadors of natural ecosystems. However, following significant declines in their populations, 11 of the 15 crane species are classified as threatened, placing them among the most endangered bird families in the world (Harris & Mirande, 2013; Krajewski et al., 2010). Their decline is primarily attributed to habitat loss (Amulike et al., 2020; Austin et al., 2018; Harris & Mirande, 2013) with numerous species struggling to obtain successful breeding outcomes as a result of breeding site loss or degradation (e.g., Fakarayi et al., 2016; Su & Zou, 2012). Yet, other crane species have shown greater resilience and noticeable population growth following increased foraging opportunities in agricultural landscapes (e.g., Fox et al., 2019; Hemminger et al., 2022; Lacy et al., 2015; van Velden et al., 2017). While the revival of these populations can be seen as a success, an increased dependence of cranes on croplands has also led to a conflict between farmers and cranes, presenting its own set of challenges (Austin et al., 2018; Hemminger et al., 2022; Nilsson et al., 2019; van Niekerk, 2018). This poses a particular concern for South Africa's endemic, small‐ranging Blue Crane (*Anthropoides paradiseus*) and the Grey Crowned Crane (*Balearica regulorum*, hereafter GCC), Sub‐Saharan Africa's most endangered crane species (Beilfuss et al., 2007; Harris & Mirande, 2013).

Despite its precarious status, the GCC, like other crane species, exhibits significant potential as an indicator species for wetland‐grassland ecosystems (Austin et al., 2018; Fraixedas et al., 2020; Han et al., 2017; Kanyamibwa, 1993). Grey Crowned Cranes generally aggregate in three kinds of groupings: pairs prior to nesting, families after hatching and flocks after fledging (Wamiti et al., 2020). Monitoring this species, especially during their breeding period (when gathered as pairs or families), is challenging as they prefer to nest in dense, inaccessible vegetation among tall reeds in wetlands and inland waterbodies (Fakarayi et al., 2016; Francis et al., 2022; Harris & Mirande, 2013; Olupot, 2016; Wamiti et al., 2020; Wen et al., 2021). The most common method for collecting breeding metrics at crane nest sites is by utilising lengthy, physical on‐foot observations to identify breeding cues followed by wading to nest sites (Wamiti et al., 2020; Wen et al., 2021). This method can be unreliable and invasive (Zelelew et al., 2019), potentially disturbing breeding activities through nest abandonment and creating direct pathways to the nest sites for natural predators (Champagnon et al., 2019; Coverdale, 2006; Francis et al., 2022; Wamiti et al., 2020, 2022). The nesting preference of GCCs and ineffective monitoring methods have resulted in major knowledge gaps in GCC ecology and reproductive success, hindering the development of effective conservation strategies.

Piloted airplane surveys are beneficial when covering large areas over short periods, which minimises the chance of repeatedly detecting individual birds or flocks and improves population estimates (Galloway‐Griesel et al., 2022; Kingsford & Porter, 2009). However, apart from being a major cause of research‐related mortalities (Sasse, 2003), aerial surveys are generally suited to larger‐sized animals, open habitats and clear weather and often require specific flight paths (Hedges & O'Brien, 2012; Marchowski et al., 2018). Financially, aerial surveys can be more costly (Anderson & Gaston, 2013) but could be more cost‐effective if on‐foot observers require payment or if the area to be monitored is difficult to access (Marchowski et al., 2018). Because of these limitations, airplane‐based monitoring of GCCs in the KwaZulu‐Natal province of South Africa is only conducted once a year. Furthermore, these surveys occur during winter, primarily focusing on monitoring the previously declining Wattled Crane (*Bugeranus carunculatus*) breeding populations (Galloway‐Griesel et al., 2022). Although this method has effectively monitored GCC population trends (as GCCs flock during winter), it does not allow for effective monitoring of this species' breeding, which takes place during the summer months.

Drones have often been demonstrated to be a more versatile and cost‐effective alternative to traditional monitoring methods (Hodgson et al., 2016; Sorrell et al., 2023). Numerous studies have reported using drones to obtain precise counts and accurate identification of breeding populations of multiple bird species (Afán et al., 2018; Hodgson et al., 2018; Lyons et al., 2018; Marchowski et al., 2018; Wen et al., 2021). Using drones to monitor breeding outcomes can also reduce the time spent around nest sites (Sikora & Marchowski, 2023). Despite their usefulness, drones can cause disturbance to animals (Duporge et al., 2021; Mulero‐Pázmány et al., 2017; Schad & Fischer, 2022; Schroeder et al., 2020), with birds being, on average, more sensitive to drone monitoring than other vertebrate types (Mulero‐Pázmány et al., 2017; Rebolo‐Ifrán et al., 2019). Guidelines for using drones to study animals usually suggest that small drone sizes (<2 kg), implementing further take‐off distances from the subject/s, higher flight heights, slow speeds and horizontal rather than vertical approaches can reduce the disturbance imposed on birds, but these responses can vary between species (Barr et al., 2020; Duporge et al., 2021; Lyons et al., 2018; Marchowski, 2021; Sorrell et al., 2023; Vas et al., 2015; Weimerskirch et al., 2018; Wilson et al., 2021). Drone use in crane research and monitoring is in its infancy, with studies generally using drones to estimate population densities (e.g., Sandhill Cranes *Grus canadensis* (Stark et al., 2017), Siberian Cranes *Leucogeranus leucogeranus* (Wen et al., 2021) and Common Cranes (*Grus grus*) (Chen et al., 2023)). Exploratory work investigating 33 bird species has shown that drones generally impart minimal disturbance when counting populations; however, the responses of Common Cranes towards drones in this study were inconclusive (Marchowski, 2021). As such, a formal investigation into whether drones can effectively monitor cranes and, if so, how they should be flown needs to be undertaken.

This study aimed to compare the behavioural responses of three GCC social groupings (pairs, families and flocks) to two monitoring methods (on‐foot approaches and drone flight heights) with the intention of reducing stress on GCCs. It also evaluated the responses of breeding birds (pairs and families) to two distinct approach angles (diagonal vs. vertical). We hypothesised that closer on‐foot distances, lower drone flight heights and vertical drone approach angles would increase disturbances to crane groupings. Finally, this study set out to determine the distances at which the probability of GCC groupings displaying evasive behaviours (e.g., walking or flying away) exceed the probability of no evasive behaviour. These findings will collectively contribute to developing appropriate monitoring guidelines for GCCs and other large bird species residing in difficult‐to‐access environments.

## 2 METHODS AND MATERIALS

### 2.1 Study area

This study took place in the southern parts of KwaZulu‐Natal, South Africa, primarily around the Underberg, Franklin and Kokstad regions. This summer–rainfall region (650–1000 mm per annum) has large areas of open grasslands and wetlands. Agriculture (intensive cropping and dairying, extensive beef and sheep) and commercial forestry (*Pinus* and *Eucalyptus*) are the primary land‐use types in these regions.

### 2.2 Experimental design

#### 2.2.1 Experiment 1: Monitoring method comparison experiment

**FIGURE 1:**
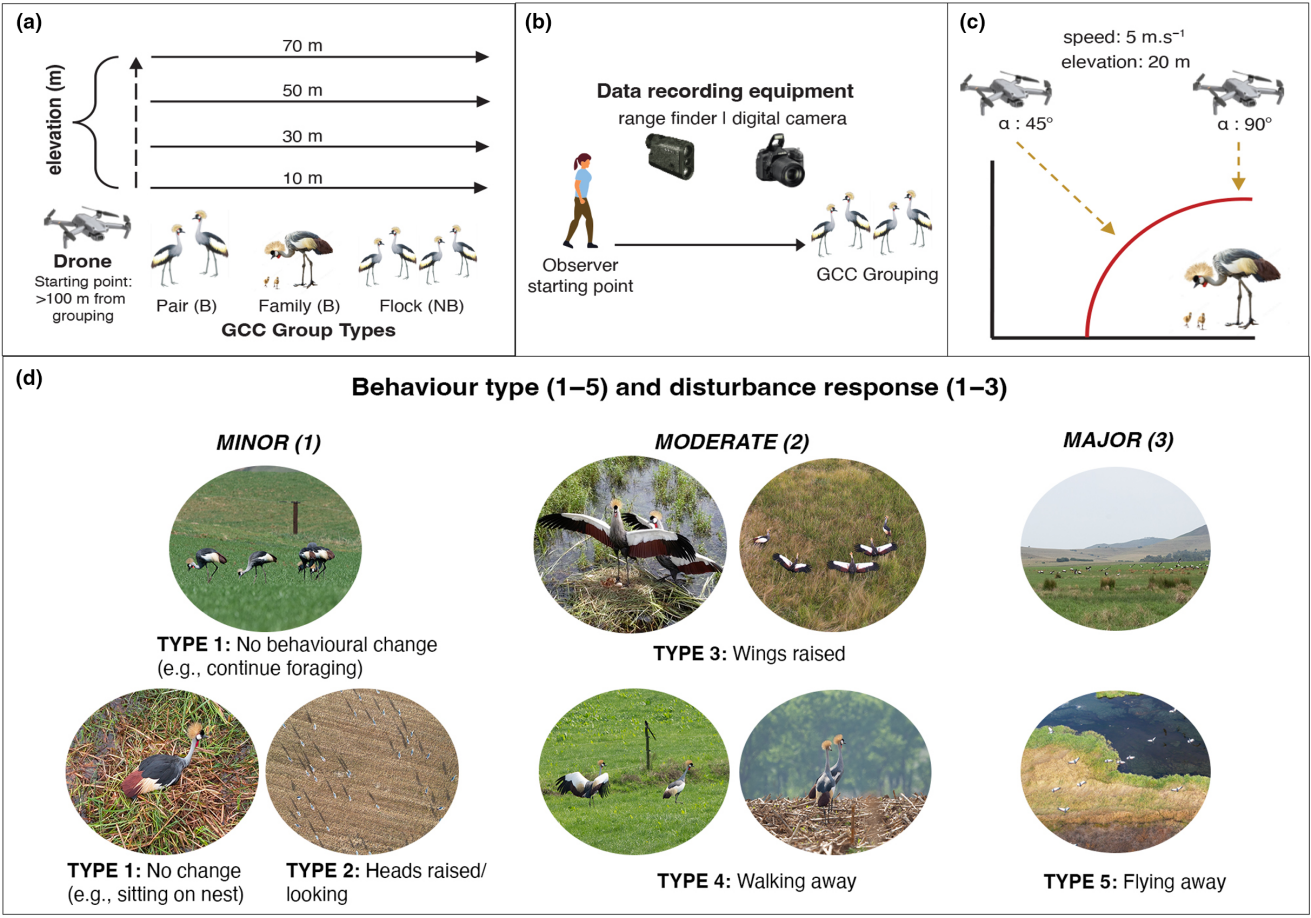
Visual depiction of (a) schematic representation of systematic drone flight paths to test bird response to the presence of a traversing drone, B = breeding and NB = non‐breeding social groupings; (b) on‐foot methodology, using a range finder to determine distance between observer and subject grouping and a digital camera to capture the behavioural responses of subject groupings as the observer approaches, (c) schematic representation of the breeding bird approach experiment and (d) behaviour response types as seen from drone and on‐foot approaches. Behaviour types 1–5 are representative of the behaviour response types used for the majority of analyses. Disturbance responses 1–3 are representative of the responses used for the approach angle investigation.

Although physiological measurements provide the ultimate indication of stress in animals and should be encouraged where possible (Geldart et al., 2022; Weimerskirch et al., 2018; Zink et al., 2023), changes in animal behaviour are often immediate (Borrelle & Fletcher, 2017) and can provide cost‐effective metrics of animal stress. Trial observations included recording the behavioural cues of GCC groupings (pairs, families and flocks) in response to either of the two monitoring methods (on‐foot, drone) across various distances and flight heights. Behavioural cues were categorised similarly to those outlined in Cantu De Leija et al. (2023) and Vas et al. (2015), alongside personal observations of GCC behaviours. These categories were as follows: no behaviour change (1), heads raised to observe surroundings (2), wings raised (3), moving away (4) and flying away (5) (Figure 1d). All trial observations were undertaken by the same observer (CRD), and care was taken to wear similarly coloured clothing for each of the trials to control for the impact that certain clothing colours can have on bird flight initiation distances in rural areas (Zhou & Liang, 2020). Upon locating a GCC grouping, the first methodology to be carried out on the grouping was chosen randomly (on‐foot or drones) and was subsequently applied to all groupings in the vicinity. If these groupings were still in the vicinity after the first method had been carried out, then the second method was undertaken on the remaining groupings.

##### On‐foot monitoring

The observer approached the group at a constant walking speed of approximately 1 m s^−1^, making a reasonable effort not to disturb the grouping (e.g., avoiding noises and sudden movements). Observations were noted at the start of each trial, every 10–15th steps thereafter, and again if any change in GCC behaviour was observed. Each observation included measuring the distance between the observer and the grouping using a range finder (Vortex Crossfire HD LRF‐CF1400 Rangefinder) and taking a photograph (Nikon D7200 with 100–400 mm Sigma lens) of the group, which enabled post hoc behaviour coding. Observations were recorded until groupings displayed a type 5 response (flying away).

##### Drone methodology

The drone pilot was positioned at least 100 m from GCC groupings before drone take‐off, as per Vas et al. (2015), and at a similar elevation to the GCC grouping. On some occasions, the pilot found it practically impossible to position herself at 100 m from the grouping. When this was the case, the drone was deployed from at least 80 m from the GCC groupings. The drone was deployed from the pilot's location to a randomly pre‐selected flight height (10, 30, 50 or 70 m above the deploy point), then flown over the grouping at the selected flight height at a speed approximating 5 m s^−1^ with video recording activated to facilitate post hoc behaviour coding (Figure 1a,d). After completing a trial, the drone was flown approximately 80 m beyond the grouping and then returned to the deployment point to avoid flying over the grouping during the return flight. All drone flights were conducted using a standard Mavic Air 2S drone (DJI Technology Co., Shenzhen, China) (595 g, 1‐inch 20 MP sensor, 8× zoom, 65 dB low noise propeller). The drone was piloted using the DJI Fly application on an iPhone 13 device (DJI, 2022).

It was challenging to determine the precise height of the drone above the grouping in situ as the drone only reports flight height relative to the take‐off point. The flight height above GCC groupings was therefore determined post hoc by extracting elevation values for the drone deployment and subject locations from a high‐resolution (±2 m) digital elevation model (GeoSmart Space, 2019) of the study area using the coordinates as recorded by the drone's Global Positioning System. The drone's actual height was therefore calculated as:

$$\text{flight height}={\text{elevation}}_{\text{deploy}}+{\text{height}}_{\mathrm{UAV}}-{\text{elevation}}_{\text{group}}$$

where flight height is the actual height of the drone above the grouping, elevation_deploy_ is the elevation above sea level of the point from which the drone was deployed, height_drone_ is the flight height reported by the drone flight log for the point at which the drone was above the grouping and elevation_group_ is the elevation above sea level for the point where the grouping was located.

Data collection from each grouping continued until a maximum of four trials had been recorded (with a 10‐min interval between trials to allow birds to return to their prior behaviour) or until the subjects flew beyond the range of the drone. As a precaution, the drone was always flown manually to allow the pilot to easily manoeuvre and control the drone to avoid any potentially aggressive behaviour from the target or non‐target species in the study area. Each site was scanned for non‐target species using binoculars before beginning a drone mission. Whilst in flight, the pilot remained aware of any new individuals of the target or non‐target species entering the site. As far as practically possible, flights over any non‐target species were avoided. If any non‐target species displayed behavioural signs of discomfort in the drone's presence (e.g., aggressive behaviour towards the drone from territorial or breeding birds or birds of prey, obvious fleeing from the environment after launching the drone demonstrating substantial fear, evidence of nesting or breeding by non‐target species) the pilot avoided flying whilst that species was near the flight route. If such behavious were observed from territorial birds, future flights at that site were terminated.

##### Post hoc behavioural coding

Video and photo footage were assessed post hoc by a single person (CRD). Each photo taken during the on‐foot monitoring experiment was considered an observation. From each video recorded during the drone monitoring experiment, the frame directly above the grouping was extracted and used as the observation. If birds responded with a type 5 response before the drone reached the grouping, the frame closest in time was used to determine the drone's position whilst focusing on the initiated flight response of the grouping. Individual birds' behaviour (type 0–5) was identified and noted at each observation, and the total number of subjects displaying each of the five behaviours was recorded.

#### 2.2.2 Experiment 2: Nesting approach experiment

Breeding behavioural cues consisted primarily of a lone crane foraging near a water body or wetland (Wamiti et al., 2020). Breeding birds were observed both during nesting (parents and chicks situated at the nest) and after nesting (parents and chicks observed away from the nest, either in reeds or foraging along the shoreline or in croplands, grasslands or pastures). Once identified, a drone flight was initiated to locate the potential nest site or the breeding birds. If either of these were found, we recorded it as an observation. At approximately 20 m from the subject, the drone was lowered to a flight height of approximately 20 m. The drone was then manoeuvred towards the subjects either by flying diagonally (an angle of approximately 45°) or vertically (flying horizontally until above the subjects and then descending at an angle of approximately 90°), slowly descending until approximately 7.5 m from the subjects. The distances reported here are approximate because of the limitations of determining distances in situ via the drone. Video recording was enabled throughout the approach to facilitate post hoc behavioural coding as follows: (1) little disturbance (either looking, remaining sitting, holding ground or standing up), (2) moderate disturbance (raising wings, walking or running away from offspring), or (3) major disturbance (flying away from offspring). A schematic representation of this method is provided in Figure 1c.

### 2.3 Statistical analyses

Individual images represented ordinal trials, and coded behavioural responses served as the independent variable. The number of birds displaying each response was then determined for each image.

Three analyses were conducted to assess the impact of either monitoring method on the behaviour of GCCs. We evaluated (1) the average distance at which a particular behavioural response was observed when monitoring non‐nesting GCCs on foot (monitoring method comparison experiment), (2) the effect of drone flight height on the behavioural scores of non‐nesting GCC group types (drone flight height experiment) and (3) the effect of approach angle on the behavioural response of nesting GCCs (nesting approach angle experiment). All analyses were conducted using R 4.2.2 (R Core Team, 2022). Post hoc comparisons were generated using the *emmeans* R package (Lenth, 2023) with *p*‐values adjusted using the Tukey method for multiple comparisons. Averages are presented as means (±95% confidence intervals) both in‐text and in figures.

#### 2.3.1 Experiment 1: Monitoring method comparison experiment

The distance at which the categorised GCC behaviours were observed between the two methods was modelled using a linear mixed effect regression (Equation 1) using the ‘lmer’ function from the *lme4* R package (Bates et al., 2015). Behavioural response (categorical with five levels), monitoring method (categorical with two levels – ‘On‐foot’, ‘Drone’) and their interaction were included as fixed effects. Subject grouping ID was included as a random effect to control for repeated measurements on distinct subject groupings (random effect LRχ^2^ = 24.35, df = 1, *p* < .001). Controlling for subject grouping also assisted in controlling for variation in start distance during the on‐foot monitoring approach. The model was weighted by the proportion of birds within the observation exhibiting the behaviour type at each distance, and the distance was log‐transformed to improve the normality of the residuals.

(1) $$\begin{array}{c} \log\left(\mu_{\mathrm{ij}}\right)={\text{Behaviour}}_{\mathrm{ij}}+{\text{Method}}_{\mathrm{ij}}+{\text{Behaviour}}_{\mathrm{ij}}\\ \times {\text{Method}}_{\mathrm{ij}}+{\mathrm{SubjectID}}_{i}. \end{array}$$

where ${\text{Distance}}_{\mathrm{ij}}$ is the jth observation of SubjectIDi, and ${\text{SubjectID}}_{i}$ is the random intercept which is assumed to be normally distributed with a variance of $\sigma^{2}$.

The second analysis considered the type of behavioural responses exhibited across group types and the distance between the observer and the subject grouping when being monitored on‐foot (Equation 2). We used a cumulative link mixed effects model (clmm) with a logit link function from the *ordinal* R package (Christensen, 2023) to model the behaviour response (an ordinal, non‐normally distributed measurement). Cumulative link models are used to handle ordinal, non‐continuous response data with the output determining the probability of each level of the response occurring. Group type (a factor with three levels; ‘Pair’, ‘Family’, ‘Flock’), distance to the subject grouping (covariate) and the interaction of these two variables were included as fixed effects. Although the inclusion of subject grouping ID as a random effect to control for both repeated measurements and differences in monitoring start distance did not significantly improve the model (LRχ^2^ = 0.418, df = 1, *p* = .518), it was included as the results were more conservative under the model with the random effect structure compared to the model without this structure.

(2) $$\begin{array}{c} \text{logit}\left({\text{Behaviour}}_{\mathrm{ijk}}\right)=\beta 0_{k}\\ \;-\left({\text{GroupType}}_{\mathrm{ij}}+{\text{Distance}}_{\mathrm{ij}}+{\text{GroupType}}_{\mathrm{ij}}\times {\text{Distance}}_{\mathrm{ij}}+{\mathrm{SubjectID}}_{i}\right) \end{array}$$

where ${\text{Behaviour}}_{\mathrm{ijk}}$ is the jth observation of the kth behaviour response type of SubjectIDi, $\beta 0_{k}$ is the threshold parameter for behaviour response type k and ${\text{SubjectID}}_{i}$ is the random intercept which is assumed to be normally distributed with a variance of $\sigma^{2}$.

A cumulative link model (clm) was used to model the behavioural responses to drone monitoring using a similar approach to that described in Equation 2 (but without the random effect structure) in the *ordinal* R package. The effect of individual subjects was considered minimal due to (1) the extended nature of these observations, (2) the random ordering of heights flown and (3) the movement of individuals between groupings and between trials, and it was therefore not recorded or incorporated into this analysis. Behaviour responses did not vary significantly due to drone deployment distance (*χ* ^2^ = 1.429, df = 1, *p* = .232), so its effect was not incorporated into the model. Drone height above the GCC grouping (covariate) and the group type being observed (a factor with three levels – ‘Pair’, ‘Family’ and ‘Flock’), together with the interaction of these two variables, were included as fixed effects. We determined the distance at which the most evasive behaviours (moving and flying away) would occur more than 50% of the time for both monitoring methods. This indicated the grouping's flight initiation distance – a standard metric used to compare stress induced through bird monitoring methods (Blumstein, 2006).

A separate clm was used to assess the behavioural response of flocks to the number of birds in the flock (covariate) and the distance to the flock (covariate) for each monitoring method (Equation 3)

(3) $$\text{logit}\left({\text{Behaviour}}_{\mathrm{jk}}\right)=\beta 0_{k}-\left({\text{FlockSize}}_{j}+{\text{Distance}}_{j}\right)$$

where ${\text{Behaviour}}_{\mathrm{jk}}$ is the jth observation of the kth behaviour response type and $\beta 0_{k}$ is the threshold parameter for behaviour response type k. Because of the smaller sample size used for on‐foot monitoring, the clmm did not converge, so the effect of flock ID was ignored. The interactions of these two fixed effects were non‐significant in both models and were therefore excluded from the final models. Test statistics for clm models are presented as *X* ^2^ values and as likelihood ratio χ^2^ (LRχ^2^) values for clmm models.

#### 2.3.2 Experiment 2: Nesting approach experiment

This experiment assessed the level of disturbance experienced by breeding GCCs at diagonal or vertical approach angles. Disturbance level was an ordinal, non‐normally distributed response variable and data collection included reoccurring observations made on the same GCC breeding subjects at specific nest sites. To account for this, we modelled the disturbance level using a clmm from the *ordinal* R package (Equation 4). Approach angle (a factor with two levels – ‘Vertical’, ‘Diagonal’) and reproductive stage (a factor with two levels – ‘During nesting’, ‘After nesting’) were included as fixed effects together with their interaction. Breeding pair ID was incorporated as a random effect to control for reoccurring observations on the same breeding subjects (LRχ^2^ = 5.435, df = 1, *p* = .0197).

(4) $$\text{logit}\left({\text{Disturbance}}_{\mathrm{ijk}}\right)=\beta 0_{k}-\left({\text{Angle}}_{\mathrm{ij}}+{\text{ReproductiveStage}}_{\mathrm{ij}}+{\text{Angle}}_{\mathrm{ij}}\times {\text{ReproductiveStage}}_{\mathrm{ij}}+{\text{BreedingPairID}}_{i}\right)$$

where ${\text{Disturbance}}_{\mathrm{ijk}}$ is thejth observation of thekth disturbance response type of BreedingPairIDi, $\beta 0_{k}$ is the threshold parameter for disturbance response type k and ${\text{BreedingPairID}}_{i}$ is the random intercept which is assumed to be normally distributed with a variance of $\sigma^{2}$.

## 3 RESULTS

In total, 313 drone flights were conducted: 110 over pairs, 66 over families and 110 over flocks. The flight time totalled 2108 min and approximated 6 min and 44 s per flight. Of 56 on‐foot approaches, 26 were to pairs, 7 to families and 23 to flocks. The mean number of birds (±95% confidence intervals) in each grouping was 2 (2, 2) for pairs, 3.66 (3.20, 4.13) for families and 34.4 (34.04, 36.08) for flocks.

### 3.1 Monitoring methodology study

**FIGURE 2:**
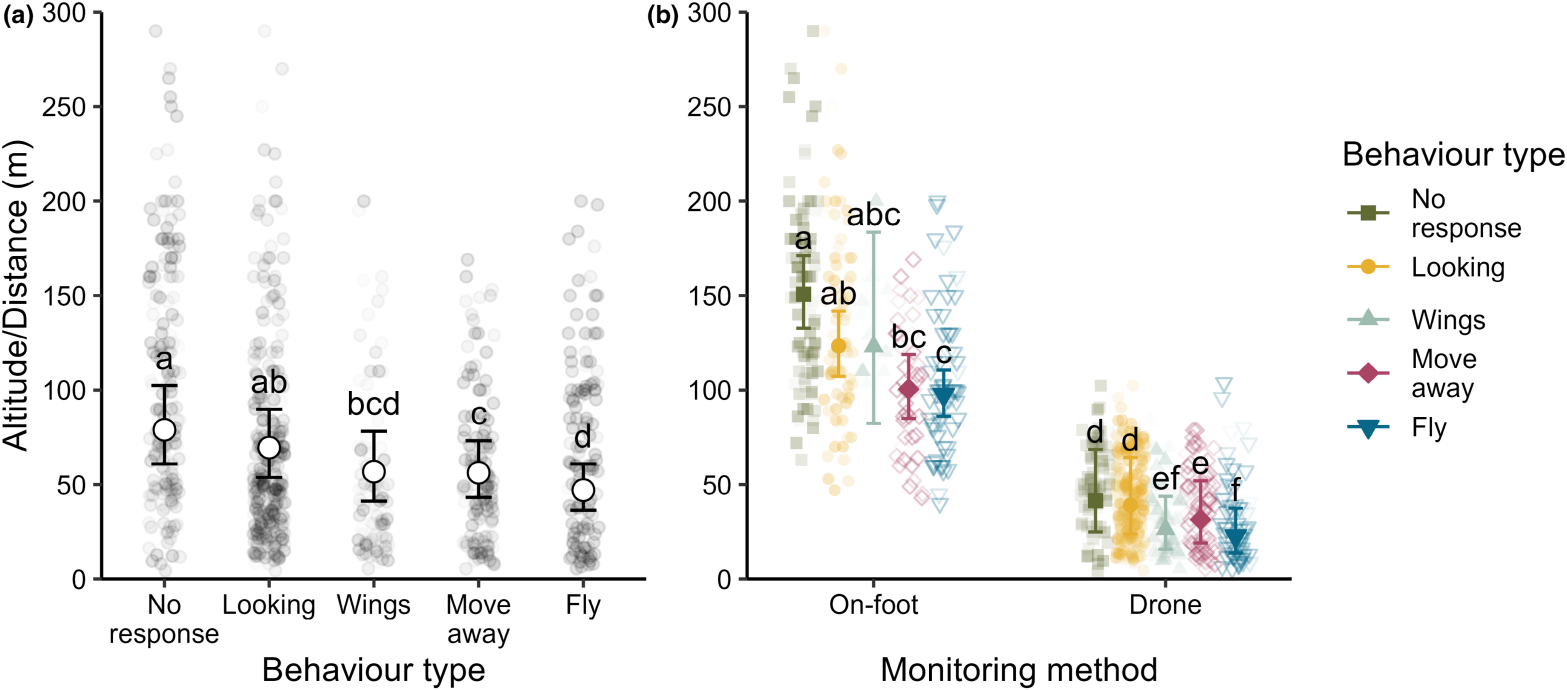
Mean (±95% confidence intervals) distance at which different behaviour responses were observed (a) for both monitoring methods and (b) for either monitoring method. Raw data points are jittered and shaded to show overlap. Points that represent a higher proportional response of a particular behaviour type are shaded slightly darker. Responses with the same letters indicate that no evidence was found for significant differences between means.

Regardless of the method used, individual birds within a grouping displayed distinct differences in their behaviour type depending on the distance of the observer or drone from the bird grouping (*F*_4,789.77_ = 23.704, *p* < .001; Figure 2a). Crane groupings showed no response at the furthest distances. As the observer moved closer to the subject/s, it was more likely that the bird/s would look, move away and finally fly (Figure 2a). Wings raised were significantly more likely to occur at closer distances than no response but often co‐occurred with looking, moving away or flying (Figure 2a). When the two monitoring methods are compared, the average on‐foot observation was recorded at 117.52 (104.89, 131.66) m from subject/s being observed, and the average of all drone flight recordings was 31.39 (19.16, 51.43) m from the observed subject/s (*F*_1,17.27_ = 29.572, *p* < .001, Figure 2b). Flight responses were initiated at 97.59 (86.13, 110.59) m when monitored on‐foot and at 22.72 (13.75, 37.52) m when monitored by drone. A significant interaction effect between the monitoring method and behaviour type indicated that the change in behaviour responses across monitoring distance was not consistent across monitoring methods (*F*_4,789.77_ = 2.858, *p* = .023; Figure 2b). The distances at which behavioural responses were recorded were more similar when recorded with a drone than on‐foot. This was primarily due to the substantial overlap in wings being raised when birds were approached on‐foot, whereas raised wings were delayed and occurred at similar distances to moving away when a drone was used.

**FIGURE 3:**
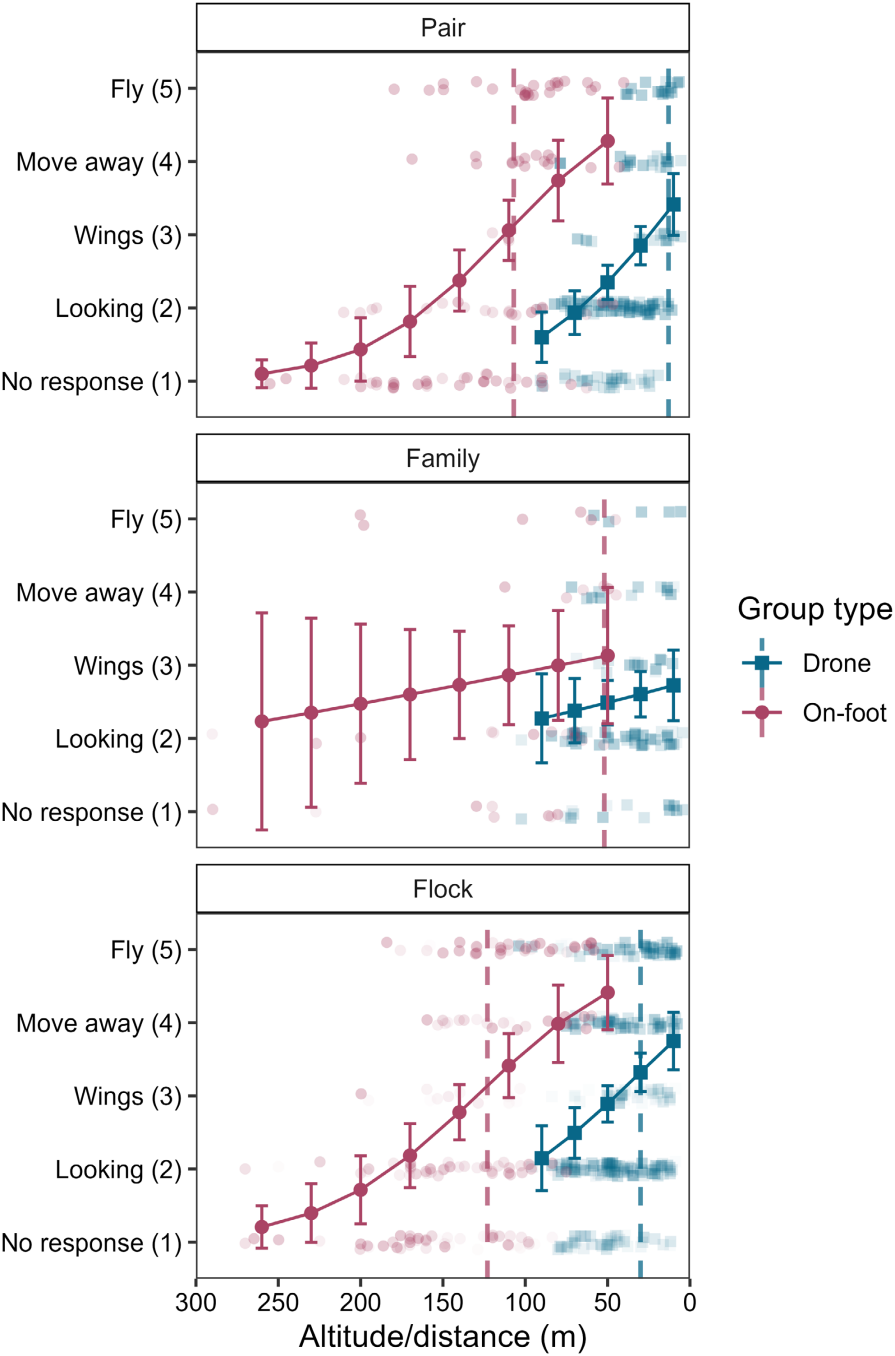
Mean (±95% confidence intervals) Grey Crowned Crane (GCC) behavioural response class observed across decreasing on‐foot approach distance and drone flight altitude for three GCC grouping types. Dashed vertical lines indicate the flight height at which the evasive behaviours (moving or flying away) become more likely to occur than non‐evasive behaviours. Drone and on‐foot results were obtained from two separate analyses due to methodological differences and so are not directly comparable. Raw data points are jittered and shaded to show overlap. Points that represent a higher proportional response of a particular behaviour type are shaded slightly darker.

On‐foot monitoring induced more evasive responses as the distance between the observer and the grouping decreased (LR*χ* ^2^ = 41.511, df = 1, *p* < .001). Evasive responses (moving or flying away) had a 50% chance of occurring at 109 m when using on‐foot monitoring. The type of social grouping did not affect the rate at which a particular behaviour response was observed (LR*χ* ^2^ = 2.431, df = 2, *p* = .297). However, the type of behavioural response changed depending on the distance from the observer between group types (LR*χ* ^2^ = 7.691, df = 2, *p* = .021; Figure 3). Families displayed no change in their behavioural responses across all distances (*Z*‐ratio = 0.825, *p* = .410), whilst both pairs (*Z*‐ratio = 3.715, *p* < .001) and flocks (*Z*‐ratio = 4.014, *p* < .001) displayed more evasive responses as the distance between the observer and the subjects decreased. The point at which evasive responses had more than a 50% chance of occurring was at 107 m for pairs, 52 m for families and 123 m for flocks.

### 3.2 Drone flight height study

As drone flight height decreased, GCC groupings were more likely to display evasive behaviours (*χ* ^2^ = 177.304, df = 2, *p* < .01; Figure 3). Considering the changes in GCC behaviours, the probability of no response and looking decreased as drone height decreased, while the likelihood of cranes moving or flying away increased (Figure 3). There was little change in the response of raising wings. The point at which evasive behaviours (moving and flying away) became more likely to occur than all other behaviour types was at 9 m.

Group types responded differently regardless of the drone's flight height (*χ* ^2^ = 34.142, df = 2, *p* < .001). Pairs and families did not differ in the type of behavioural response, with the mean behaviour class observed being between looking and raising wings (mean score = 2.55 and 2.54, respectively; *Z*‐ratio = 0.063, *p* = .998). However, flocks generally displayed raised wings (mean score = 3.07) and were significantly more likely to evade the drone than were pairs (*Z*‐ratio = 3.162, *p* = .005) or families (*Z*‐ratio = 2.861, *p* = .012).

**FIGURE 4:**
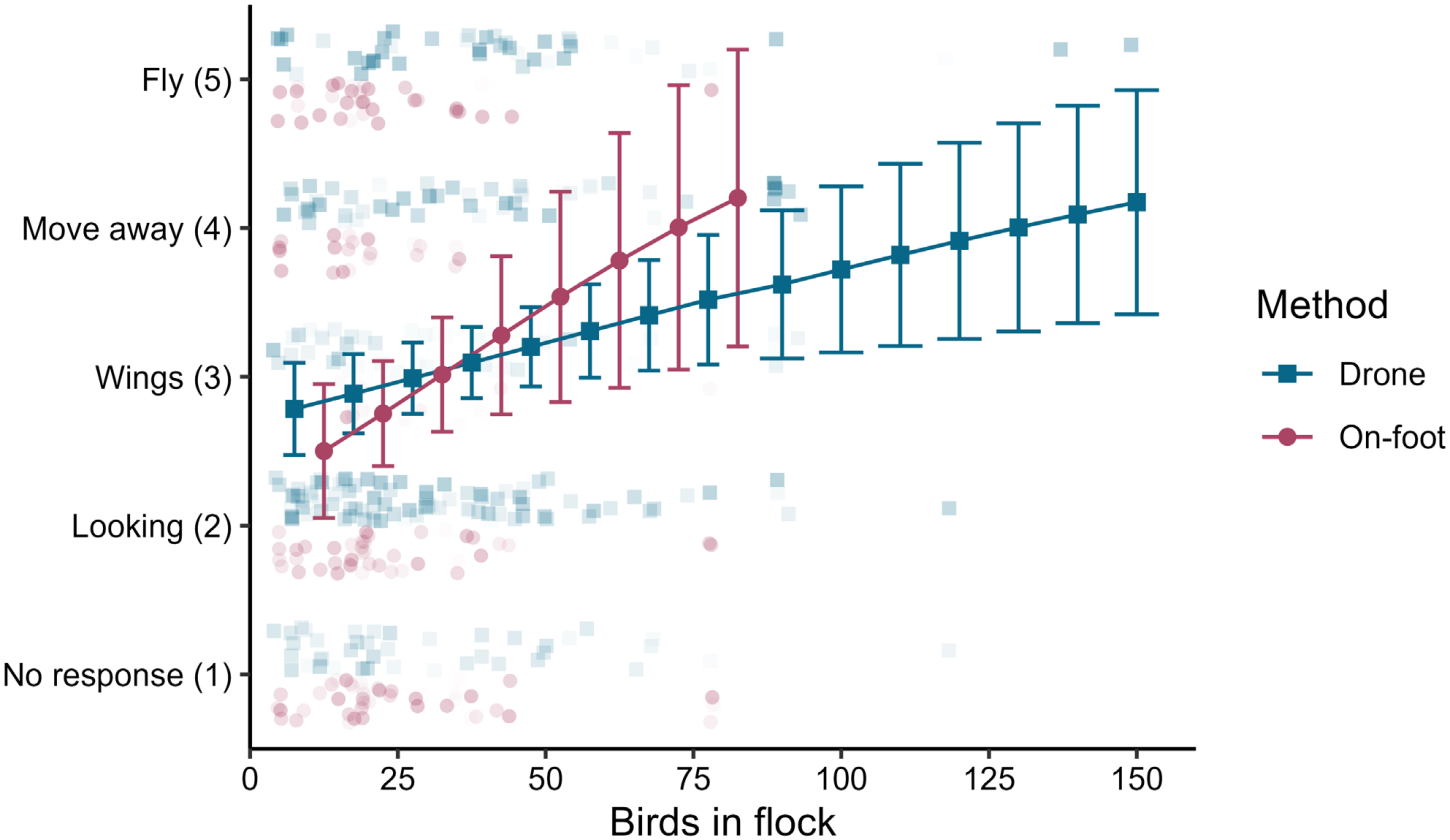
Mean (±95% confidence intervals) behaviour response class response for Grey Crowned Cranes across flock size. The methods plotted are the result of separate statistical models. Different ranges in flock size reflect the actual range of flock sizes observed when monitoring with either method. Raw data points are jittered and shaded to show overlap. Points that represent a higher proportional response of a particular behaviour type are shaded slightly darker.

Grey Crowned Crane group types responded differently to variations in drone flight heights (*χ* ^2^ = 95.175, df = 2, *p* < .001; Figure 4). Families showed no change in their behavioural responses across all flight heights (*Z*‐ratio = 0.923, *p* = .356), whilst both pairs (*Z*‐ratio = 4.571, *p* < .001) and flocks (*Z*‐ratio = 3.720, *p* < .001) showed more evasive responses as the drone flight height decreased. Pairs displayed evasive behaviour at 13 m, whilst flocks displayed evasive behaviour at 30 m. Evasive responses were consistently less likely to occur than non‐evasive responses for families across the range of drone flight heights used in this study.

There were significant changes in behaviour associated with distance to the flock and the number of birds in the flock across both monitoring methods. Reduced distance to flocks (on‐foot: *χ* ^2^ = 23.572, df = 1, *p* < .001; drone: *χ* ^2^ = 12.526, df = 1, *p* < .001; Figure 4) and increased flock size (on‐foot: *χ* ^2^ = 4.300, df = 1, *p* = .038, drone: *χ* ^2^ = 5.801, df = 1, *p* = .016; Figure 4) increased the chance of evasive behaviours across both monitoring methods.

### 3.3 Nesting approach study

**FIGURE 5:**
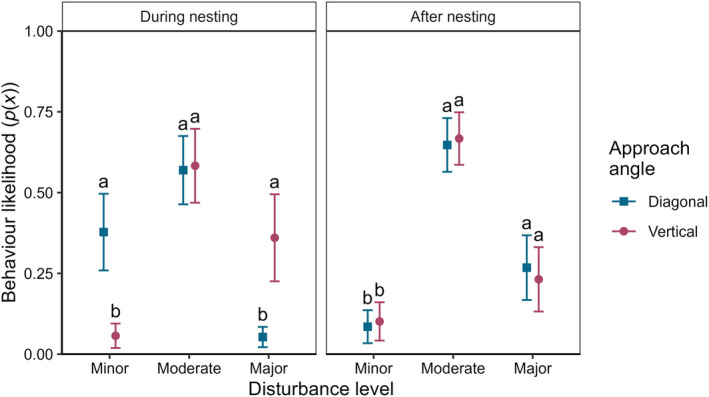
Mean probability (±95% confidence intervals) of perceived level of disturbance imposed on breeding Grey Crowned Cranes during and after nesting across two drone approach angles. Letters indicate the response of post hoc analyses conducted between approach angles for each disturbance level within breeding stage. Responses within each panel with the same letters indicate that no evidence was found for significant differences between their means.

Behavioural responses of GCCs were significantly related to drone approach angles to nests or families (LR*χ* ^2^ = 13.989, df = 1, *p* < .001; Figure 5), with vertical approaches causing a greater disturbance. There was also a difference in the type of responses observed between breeding stages (LR*χ* ^2^ = 9.032, df = 1, *p* = .003), with more evasive responses being observed during nesting (difference in mean class = 0.167 ± 0.07 standard errors). The interaction of these two factors (LR*χ* ^2^ = 9.032, df = 1, *p* = .003) showed that different responses to the approach angle occurred during nesting (Figure 5).

## 4 DISCUSSION

The findings of this study confirm the usefulness of drones for monitoring wetland‐nesting species and provide valuable species‐specific guidelines for monitoring GCCs. By conducting a total of 313 drone flights over three GCC social groupings (pairs, families and flocks), we found that all groups displayed disturbance cues earlier when approached on‐foot compared to when using a drone (Figure 2). Flocks experienced minimal disturbance when the drone was flown above 30 meters. However, larger flocks demonstrated a higher probability of evasive responses to either of the monitoring methods. Nesting birds were sensitive to the angle at which a drone approached their nest site, but after hatching, family groupings displayed the least likelihood of evading lower flight heights. Apart from addressing important practical considerations relating to improving the monitoring of the focal species, the experiments we conducted also allowed us to explore several ethological theories, which are elaborated upon below.

### 4.1 Reaction of GCC groupings to monitoring methods

#### 4.1.1 Distance and monitoring methods

Bird body mass is positively correlated with flight initiation distance, suggesting that larger birds flee from potential threats at greater distances because they require a longer take‐off (Møller et al., 2016). Large‐sized South African waterbirds follow this principle, displaying a flight initiation distance of approximately 100 m when approached on‐foot (Coetzer & Bouwman, 2017). However, one might assume that the close association of cranes with agriculture has habituated them to human disturbances, making them less sensitive to on‐foot monitoring approaches (Okes et al., 2008; Samia et al., 2015; Weston et al., 2020). Nevertheless, Black‐necked Cranes (*Grus nigricollis*) flee observer approaches at 88.33 m (Kong et al., 2021), and our findings revealed that GCCs initiated flights at 97.59 m. Therefore, GCC groupings are equally tolerant of on‐foot approaches as their local counterparts. These flight distances also suggest that cranes are sensitive to human disturbances (Coverdale, 2006; Végvári et al., 2011; Wang et al., 2011), yet most crane species are still monitored using on‐foot methods. Monitoring GCCs with drones substantially reduced their average flight initiation distance to 22.7 m. This distance was 4.29 times closer than what on‐foot monitoring could achieve. Thus, when monitoring from equal distances one can assume that drones impart less disturbance than traditional monitoring methods.

#### 4.1.2 Response of flocks to monitoring methods

Flocks demonstrated evasive behaviours sooner than the other social groupings, regardless of the monitoring method used. While several potential explanations could account for their alarmed response, we discuss here three of the more frequently encountered explanations. Firstly, this finding may support the vigilance hypothesis, which states that flocks with more individuals have an improved probability of detecting threats, which further escalates the likelihood of evasive flight behaviours (Morelli et al., 2019). Secondly, a flock's vigilance may depend on the type of habitat they utilise and the number of threats associated with that habitat. Agricultural areas, for example, are known to attract opportunistic predators (Drouilly et al., 2018). Hooded Cranes (*Grus monacha*) gathering in rice paddies surrounded by human activities display higher vigilance in these areas compared to their natural habitats (Li et al., 2015). Therefore, it is perhaps not surprising to note a similar response among GCCs that gather in harvested crop fields and pastures. Others explain that birds inhabiting agricultural landscapes must learn to identify deviations from a predator or human's routine behaviour (Samia et al., 2015). Furthermore, they suggest that such deviations in a bird's surroundings usually re‐elicits a cautious or evasive response to avoid possibly lethal threats. And thirdly, GCC flocks contain numerous younger, non‐breeding juveniles. These individuals may be more sensitive to anthropogenic disturbances because of inexperience. When juveniles are present, Black‐necked Crane flocks increase their vigilance time, likely to compensate for a lack of experience among younger cranes (Xu et al., 2013). Flocks consisting of younger birds can also display ‘false alarm flighting’ as practice to prepare for encountering a real threat (Root‐Bernstein, 2021). Monitoring GCC flocks, which often have a higher proportion of juveniles, should thus be done with care whilst maximising the distance or flight height to reduce their flight probability.

#### 4.1.3 Response of breeding birds to monitoring methods

Before employing new monitoring methods, it is essential to carefully consider any possible disturbances to a species' breeding behaviour and their environment (Cantu De Leija et al., 2023; Coverdale, 2006; Francis et al., 2022; Hodgson et al., 2018; Wamiti et al., 2022; Zink et al., 2023). The nesting phase is the most vulnerable period for many threatened ground‐breeding birds (Assersohn et al., 2021). Findings indicate that half of Whooping Crane (*Grus americana*) chick mortalities occur during hatching and before the chicks are 1 month old (King et al., 2013), while the hatching rate of GCCs is also low (Gichuki, 2000). While any approach to a nest site or family grouping for monitoring purposes likely causes some level of disturbance, researchers should note the response of the parents, which may vary depending on the perceived level of danger to either themselves or their offspring (Dowling & Bonier, 2018; Lima, 2009). For example, Piping Plover (*Charadrius melodus*) parents flee their nest at greater distances when the approaching subject is a dog in contrast to approaching humans or vehicles, since the latter is likely perceived as less threatening (Dowling & Bonier, 2018; Jorgensen et al., 2016).

Nesting GCCs generally showed increased vigilance monitoring with either method. Although drones could obtain closer distances, birds tended to flee from nest sites when approached vertically (tactics often employed by aerial predators – Vas et al., 2015) as opposed to diagonally. Cranes leave the security of their nest just days after hatching and undertake substantial movements with their young (Veltheim et al., 2019). During this period, parents were less sensitive to approach angles and were less likely to leave their chicks. When approached by either method at closer distances, GCC parents called, hopped and raised their wings (with younger chicks hiding under their parents' wings). These behaviours allude to their instinctive priority to protect their offspring by distracting predators from their offspring or to increase their perceived size as a predation deterrent (Gallego & Sarasola, 2021; Humphreys & Ruxton, 2020). Their behaviour during and after nesting thus appears to align with the parental theory, which suggests a positive correlation of parental defence with offspring age (Boucher, 1977). When applied to this species, this would mean that incubating cranes likely prioritise their own safety over the success of their eggs. However, they appear to undergo a switch once eggs have hatched since the probability of successfully rearing young increases after hatching (similar findings shown by Ge et al., 2011; Kong et al., 2021).

### 4.2 Species‐specific guidelines and recommendations

While drones may emit some disturbance to GCC groupings and collisions of a drone and target or non‐target species are possible, the latter is rare and did not occur once during the 313 flights of this study (similar results have been obtained by Marchowski, 2021). The risk of disturbance and collisions from drones should thus be placed within the context of the risks and inefficiencies of on‐foot monitoring. For example, monitoring tern nests with drones is 2.89 times faster than on‐foot observations (based on information provided by Valle & Scarton, 2021). Also, if Stork (*Ciconia Ciconia*) parents leave their nests during monitoring events, their return time to the nest is shorter when monitored with drones than on‐foot monitoring (Zbyryt et al., 2021), further emphasising the reduction in stress from drone monitoring. Although we did not explicitly measure return timing, informal observations suggest similar trends when monitoring GCCs with drones. In summary, the discussed findings thus far indicate clear advantages in utilising drones for GCC monitoring and guidelines for doing so will be expanded upon below. Although drones have many benefits, they do require greater postprocessing to extract data from the images or videos (Gonzalez et al., 2016). When considering vulnerable species that are sensitive to human disturbances, the trade‐off of longer postprocessing times in exchange for shorter exposure times may well be worth it.

#### 4.2.1 Monitoring flocks with drones

Drones are often used to monitor flocks for census purposes (Hodgson et al., 2018; Marchowski, 2021; Valle & Scarton, 2020; Wen et al., 2021). However, in this study, flocks responded negatively to flight heights below 30 m. Drones should then be flown at elevated flight heights over flocks to avoid evasive responses. Censuses typically require flying at greater altitudes (>50 m) to capture more individuals in the frame, so there is little trade‐off in higher flight heights over this group type. Notably, using drones as an alternative to annual airplane surveys would serve as a more regular and cost‐effective method to obtain valuable data which can be used to inform this species' conservation strategies.

#### 4.2.2 Monitoring breeding birds with drones

Drones were very effective in monitoring GCC breeding pairs and family groupings. To capture breeding activities clearly, we suggest monitoring nest sites after early mornings and before late afternoons (Demmer, personal observation). Once a potential nest site has been located, the drone should be manoeuvred in a way that minimises the angle of approach to minimise the parents' escape probability. Researchers should also note that displaying no behavioural response to a disturbance stimulus does not necessarily mean that the subject is not stressed, since stress may manifest through physiological responses instead (Zink et al., 2023). As such, we discourage unnecessarily disturbing incubation and parental activities (both of which are energetically costly to the parents – Geldart et al., 2022) and suggest limiting flights during these initial breeding stages, as parents are more likely to leave the nest. Higher flights and using the sensor's digital zoom capabilities can help reduce disturbances and the chance of parents leaving their offspring unattended. Although parents showed no behavioural change in response to drone approach angles after nesting, we suggest continued caution when flying at closer distances and suggest using diagonal approaches throughout all flights over pairs and families.

Future studies should also employ regular monitoring, especially when tracking families with older chicks at wetlands (as opposed to human‐constructed waterbodies) since these waterbodies often have multiple nest sites, which can lead to confusion and inaccurate data capturing. Families with fledged chicks travelled further (Thompson et al., 2022; Wolfson et al., 2020) and were sometimes more easily located by surveying the area with a vehicle instead of a drone. In some scenarios, attaching GPS bands has proven useful in monitoring Brolga Crane chicks (*Antigone rubicunda*) with minimal fatalities (Veltheim et al., 2019), but this was not the focus of our study.

### 4.3 Conclusion

This study illustrates that GCCs display differential responses according to their perceived risk of the threat (on‐foot or drone approaches). Whilst on‐foot monitoring methods remain effective when subjects are conspicuous, drones are more efficient and accurate for counting individuals in flocks, identifying nest site locations and conducting egg and chick counts. The inclusion of drones as a monitoring tool for GCCs should thus be dependent on the aim of the study and budget requirements (equipment costs and legislative requirements). Our results corroborate those of existing drone methodology studies, which suggest that it is not simply the employment of drones but how they are employed that makes them an effective data collection tool (Lyons et al., 2018; Mo & Bonatakis, 2022; Vas et al., 2015; Weston et al., 2020). Whilst this study may provide comprehensive guidelines for the research and monitoring of other large, threatened and difficult‐to‐study waterbirds, utilising drones for wildlife monitoring is highly species‐specific (Vas et al., 2015; Weimerskirch et al., 2018; Weston et al., 2020) and we caution that future studies should first develop species‐specific protocols before implementing drone methodologies.

## AUTHOR CONTRIBUTIONS

**Carmen R. Demmer:** Conceptualization (equal); data curation (equal); formal analysis (equal); funding acquisition (equal); investigation (equal); methodology (equal); project administration (equal); resources (equal); validation (equal); visualization (equal); writing – original draft (equal); writing – review and editing (equal). **Stuart Demmer:** Conceptualization (equal); formal analysis (equal); investigation (equal); methodology (equal); visualization (equal); writing – review and editing (equal). **Trevor McIntyre:** Conceptualization (equal); investigation (equal); methodology (equal); project administration (equal); resources (equal); supervision (equal); validation (equal); writing – original draft (equal); writing – review and editing (equal).

## CONFLICT OF INTEREST STATEMENT

The authors declare no conflict of interests.

## Supporting information


Data S1.

## Data Availability

The data that support the findings of this study are avaiable in Data S1 in the Supporting Information section.
